# Signatures of photo-aging and intrinsic aging in skin were revealed by transcriptome network analysis

**DOI:** 10.18632/aging.101496

**Published:** 2018-07-18

**Authors:** Byuri Angela Cho, Seong-Keun Yoo, Jeong-Sun Seo

**Affiliations:** 1 Gong Wu Genomic Medicine Institute, Seoul National University Bundang Hospital, Seongnam, Republic of Korea; 2 Department of Biomedical Sciences, Seoul National University Graduate School, Seoul, Republic of Korea; 3 Genomic Institute, Macrogen Inc., Seoul, Republic of Korea; * These authors contributed equally to this work.

**Keywords:** RNA sequencing, Genotype-Tissues Expression project, suprapubic, lower leg, intrinsic-aging, photo-aging

## Abstract

There are various factors that alter physiological characteristics in skin. Elucidating the underlying mechanism of transcriptional alterations by intrinsic and extrinsic factors may lead us to understand the aging process of skin. To identify the transcriptomic changes of the aging skin, we analyzed publicly available RNA sequencing data from Genotype-Tissue Expression (GTEx) project. GTEx provided RNA sequencing data of suprapubic (n=228) and lower leg (n=349) skins, which are photo-protected and photo–damaged. Using differentially expressed gene analysis and weighted gene co-expression network analysis, we characterized transcriptomic changes due to UV exposure and aging. Genes involved in skin development such as epidermal differentiation complex component (SPRR and LCE families), vasculature development (*TGFBR1*, *TGFBR2*, *TGFBR3*, *KDR*, *FGF2*, and *VEGFC*), and matrix metalloproteinase (*MMP2*, *MMP3*, *MMP8*, *MMP10*, and *MMP13*) were up-regulated by UV exposure. Also, down-regulated lipid metabolism and mitochondrial biogenesis were observed in photo-damaged skin. Moreover, wound healing process was universally down-regulated in suprapubic and lower leg with aging and further down-regulation of lipid metabolism and up-regulation of vasculature development were found as photo-aging signatures. In this study, dynamic transcriptomic alterations were observed in aged skin. Hence, our findings may help to discover a potential therapeutic target for skin rejuvenation.

## Introduction

Skin is the largest organ of human body and its physiological changes are affected by intrinsic or extrinsic factors [1,2]. Aging is a biological process which occurs to every individual from the moment of one’s birth and it is one of “the factor” that causes changes in skin. In addition, among various factors, ultraviolet (UV) light is the most well-known extrinsic factor which enhances skin aging [1]. Majority of the organs are located internally in human body, however, in case of skin, it holds both sides of the coin; some parts are UV protected and some other parts are UV exposed.

There are a few previous studies regarding the aging-related gene expression changes in skin. Glass *et al.* examined the gene expression changes with age in various human tissues including skin [3]. They only suggested that a significant proportion of aging-related changes in gene expression profile are tissue specific. Furthermore, Yang *et al.* studied the aging-related gene expression alteration in multiple tissues [4]. However, in their study, skin did not show the aging-related changes. In addition, Kaisers *et al.* also reported that the aging-related gene expression changes were not displayed in skin [5]. Conversely, a study done by Kimball *et al.* demonstrated a few changes due to aging in skin [6]. In their study, down-regulated mitochondrial function was illustrated as one of the signatures of aged skin. Additionally, increased cytokine production and immune responses with aging were also described.

As mentioned above, although there are a few studies on the aging-related gene expression changes in skin, the results are still debatable. Moreover, a large-scale study on the gene expression changes of skin tissue according to UV exposure has not been reported yet. Therefore, elucidating the consequences of intrinsic- and photo-aging in transcriptomic level will expand our understanding on skin aging.

In the present study, we used RNA sequencing data of skin from Genotype-Tissues Expression (GTEx) project [7,8]. GTEx database provided a large number of transcriptome data of skin samples from two different origins, suprapubic (UV protected) and lower leg (UV exposed), which enabled us to identify the transcriptomic shift of skin tissues due to UV exposure and aging.

## RESULTS

### DEG analysis of gene expression changes upon UV exposure

**Figure 1:**
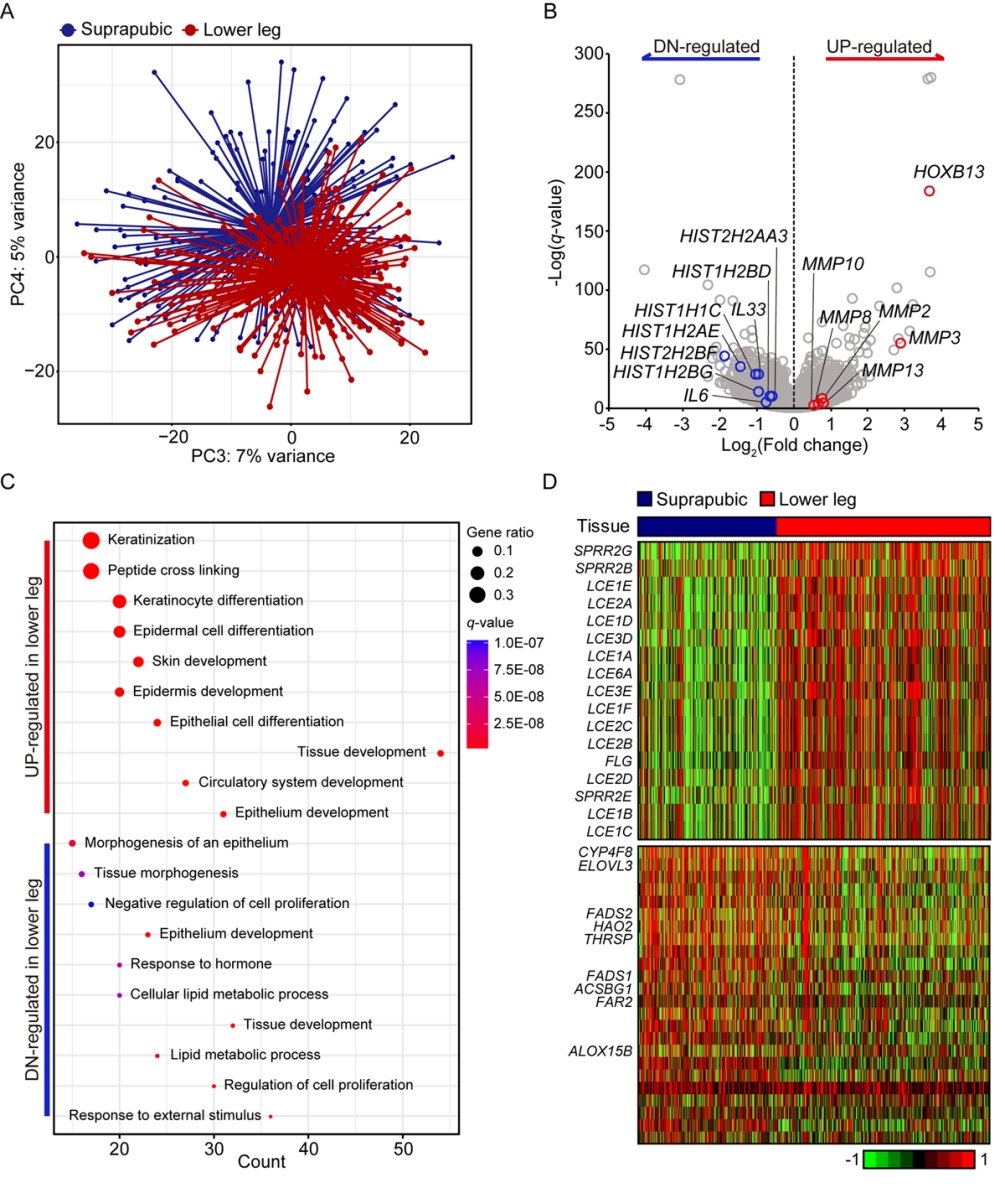
**Transcriptome analysis of lower leg (UV exposed) and suprapubic (UV protected) skin samples.** (**A**) Distinct separation of two groups was observed with principal component analysis. (**B**) Differentially expressed genes were displayed on the volcano plot. A few notable genes were marked. (**C**) Using differentially expressed genes, enriched pathways were shown in dot plot. Top 10 the most enriched pathways were used. Gene ratio = no. of genes that were enriched on the given pathway/ total no. of genes on the given gene set. *q*-value illustrated the significance. (**D**) Gene expression profiles of epidermal differentiation complex component genes (upper panel) and lipid metabolic process related genes (lower panel).

Principle component analysis revealed clear transcriptomic difference between suprapubic and lower leg tissues, possibly due to UV exposure (Figure 1A). Moreover, DEGs between two groups also showed the evident distinction of two tissues in transcriptome level (Figure 1B). Down-regulated DEGs included *IL6* and *IL33*, which are involved in wound heading [9–11], in addition to several histone genes such as *HIST2HBF*, *HIST1H2AE*, *HIST1H1C*, *HIST1H2BG*, *HIST1H2BD*, and *HIST2H2AA3*. The down-regulation of histone genes by external stimuli, which corresponds to our result, was previously reported [12]. This might suggest the decreased histone genes in lower leg was affected by chronic UV exposure. In addition, *HOXB13*, plays a role in fetal skin development and cutaneous regeneration [13], and several matrix metalloproteinase (MMP) genes including *MMP3*, *MMP13*, *MMP2*, *MMP8*, and *MMP10* were elevated in lower leg. In relation to MMP genes, their up-regulation in photo-damaged skin is well-reported event which induces collagen fragmentation [1,14].

Using pathway enrichment analysis, the functional characteristics of up- and down-regulated genes were discovered (Figure 1C). Especially for the up-regulated genes, they were mostly enriched in skin/tissue development. In epidermis, a gene complex called the epidermal differentiation complex (EDC) regulates skin barrier function and it is composed of small proline-rich (SPRR) proteins, late cornified envelope (LCE) proteins, S100 family, and S100 fused type protein (SFTP) family [15]. SPRR genes were initially identified as UV responsive genes and LCE genes share the same property [16]. Here, 13 LCE genes were increased as well as three SPRR genes and one SFTP family gene upon UV exposure (Figure 1D).

In case of down-regulated DEGs, they were mainly enriched in ‘response to external stimulus’, ‘regulation of cell proliferation’, and ‘lipid metabolic process’. Some of lipid metabolic genes discovered in our analysis were also reported as declined genes in patients with psoriatic (*ACSBG1*, *ALOX15B*, *ELOVL3*, *FADS1*, *FADS2*, and *THRSP*) [17] or atopic dermatitis (*CYP4F8*, *ELOVL3*, *FADS1*, *FADS2*, *FAR2*, and *HAO2*) [18] (Figure 1D). Additionally, *LCE3D*, *SPRR2B*, and *SPRR2G* which were reported as up-regulated in psoriatic skin were also significantly increased in UV exposed lower leg in our analysis [17]. These results might suggest the increased susceptibility to skin disorders with UV exposure.

### Gene co-expression network changes by UV exposure

Based on the above results, we hypothesized that UV exposure induces dramatic gene expression changes in skin, hence, performed in-depth subsequent comparison analysis between suprapubic and lower leg using WGCNA. Transcriptome profile was analyzed with constructed gene co-expressed networks that represent biologically related genes.

**Figure 2:**
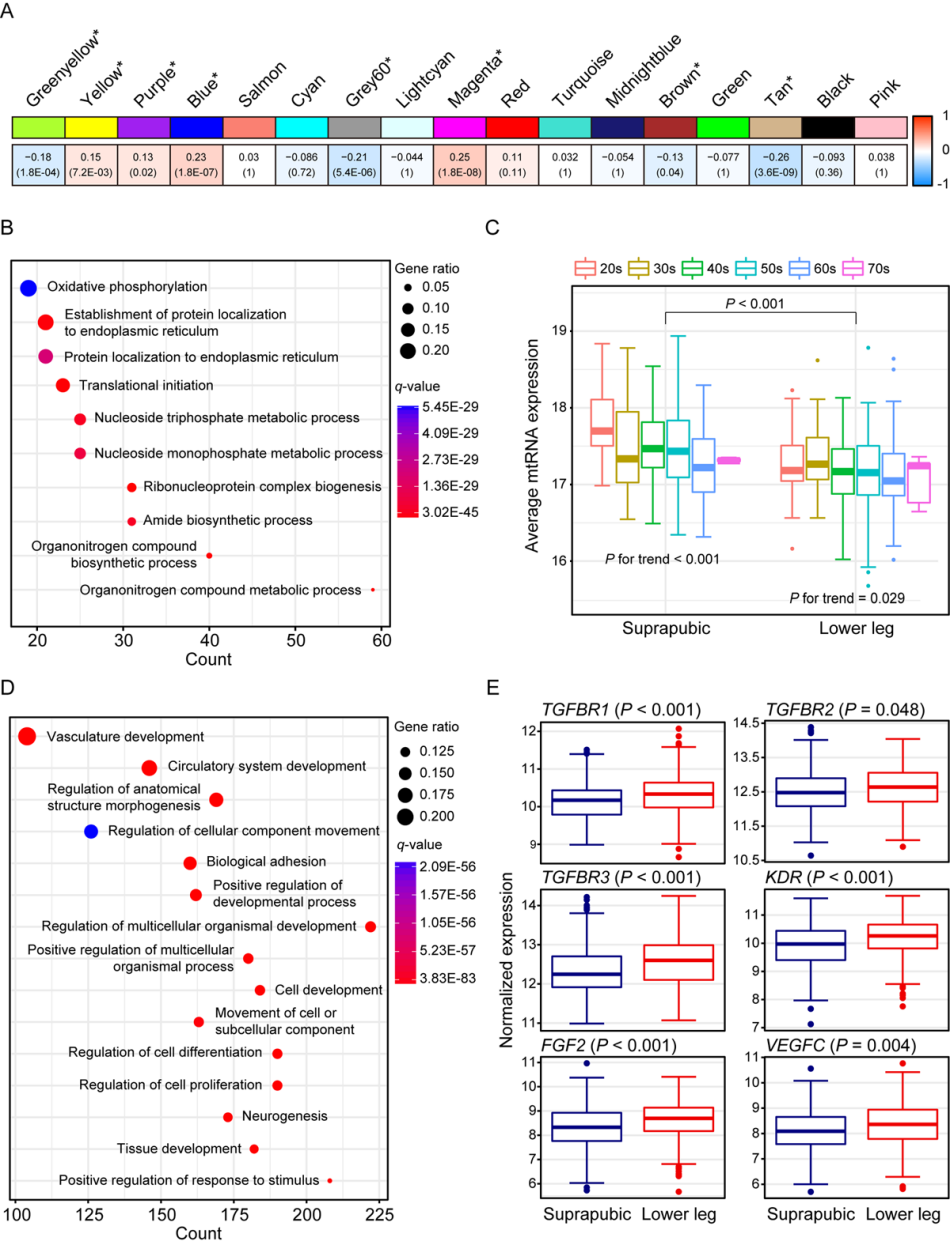
**Weighted gene co-expression network analysis using all samples.** (**A**) Module trait relationship of constructed modules. The statistically significant modules were marked with asterisk. The correlation values and Bonferroni-corrected *P*-values (in the bracket) were marked. (**B**) Pathway enrichment result for tan module. Top 10 the most enriched pathways were shown. (**C**) Estimated mitochondrial DNA copy number using mitochondrial RNA (mtRNA) expression level. Both tissues showed down-regulation of average mtRNA expression with aging (suprapubic: *P* for trend < 0.001 and lower leg: *P* for trend = 0.025). (**D**) Pathway enrichment result for blue module. Top 15 the most enriched pathways were used. (E) Relative gene expression levels of six angiogenesis related genes from blue module.

We identified 17 modules with UV exposure, eight statistically significant modules were constructed, and top 10 most correlated genes from each module are listed on Supplemental Table 1. The most significant module, tan (R = -0.26 and Bonferroni-corrected *P* = 3.6E-09), showed down-regulation with UV exposure (Figure 2A). Tan module was related to ‘oxidative phosphorylation’, ‘protein localization’, and ‘organonitrogen compound biosynthetic/metabolic processes’ (Figure 2B). ‘Oxidative phosphorylation’ included numerous genes associated with mitochondrial function such as NADH dehydrogenase (e.g. *NDUFA1* and *NDUFB1*) and ATP synthase (e.g. *ATP5C1*), and cytochrome c oxidase (e.g. *COX6C* and *COX7C*). Moreover, organonitrogen compound related pathways contained genes associated with mitochondrial ribosomal proteins (e.g. *MRPL22* and *MRPL52*). Down-regulation of such genes may represent declined mitochondrial function which is previously reported as one of the signatures of aging across multiple human tissues [4,6,19]. To elucidate this phenomenon further, we analyzed mitochondrial DNA (mtDNA) copy number changes as it is closely linked with mitochondrial biogenesis [20]. Since mtRNA expression and mtDNA copy number are highly correlated [19,21], we estimated mtDNA copy number using mtRNA expression. As a result, UV induced mtDNA copy number defect was discovered (Figure 2C, Mann Whitney test *P* < 0.001). Additionally, a trend of decrement was observed with aging in both tissues (suprapubic: *P* for trend < 0.001 and lower leg: *P* for trend = 0.029).

There were three other significantly down-regulated modules with UV exposure: grey60 (R = -0.21 and Bonferroni-corrected *P* = 5.4E-06), greenyellow (R = -0.18 and Bonferroni-corrected *P* = 1.8E-04), and brown (R = -0.13 and Bonferroni-corrected *P* = 0.04) (Supplemental Figure 1). Genes from grey60 module were mostly enriched with lipid metabolic related processes and showed decrement which was also observed in previous results [22,23]. In particular, top 10 most correlated genes from Grey60 included three fatty acid related genes such as *FADS2*, *ELOVL3*, and *FADS1* (Supplemental Table 1). Down-regulated ‘cellular response to stress’, ‘RNA/mRNA process’, ‘protein localization’, and ‘cell cycle’ were also exhibited.

The second most correlated module, magenta (R = 0.25 and Bonferroni-corrected *P* = 1.8E-08), was associated with ‘skin development’, ‘keratinocyte differentiation’, and ‘keratinization’ (Supplemental Figure 1). In parallel with results from DEG analysis, many EDC components were elevated including LCE genes. LCE proteins function in the last step of cornification [15] and the cornified cells consist degraded organelles and eventually get detached from the skin layer [24–26]. The altered cornified envelope by UV exposure may induce a reduced barrier function of skin. Moreover, *FLG2*, one of well-known EDC genes, is found as top 10 most correlated genes (Supplemental Table 1) which again emphasized that genes from magenta module contributes to skin development pathway.

Wrinkle is the most well-known characteristic of aged skin and it is reported that angiogenesis is closely associated with its generation [27–30]. Blue module (R = 0.23 and Bonferroni-corrected *P* = 1.8E-07) was enriched with various biological processes including ‘vasculature development’, ‘circulatory system development’, and ‘regulation of anatomical structure morphogenesis’ (Figure 2D). Several genes that are involved in angiogenesis including *FGF2*, *KDR*, *TGFBR1*, *TGFBR2*, *TGFBR3*, and *VEGFC* were significantly increased in lower leg relative to suprapubic (Figure 2E).

In addition, yellow (R = 0.15 and Bonferroni-corrected *P* = 7.2E-03) and purple (R = 0.13 and Bonferroni-corrected *P* = 0.02) also displayed up-regulation in lower leg (Supplemental Figure 1). Many collagen genes were included in up-regulated modules including blue, yellow and purple. Collagen is a well-known component of skin layer and plays an important role in skin structure and, usually, collagen degradation is frequently observed in photo-damaged skin [31]. However, in our analysis, increment of those genes was exhibited, thus, we could assume that increased collagen genes can also alter skin structure upon UV exposure as well as degraded collagen genes [32] (Supplemental Table 3).

### Aging associated gene expression changes in UV exposed skin

**Figure 3:**
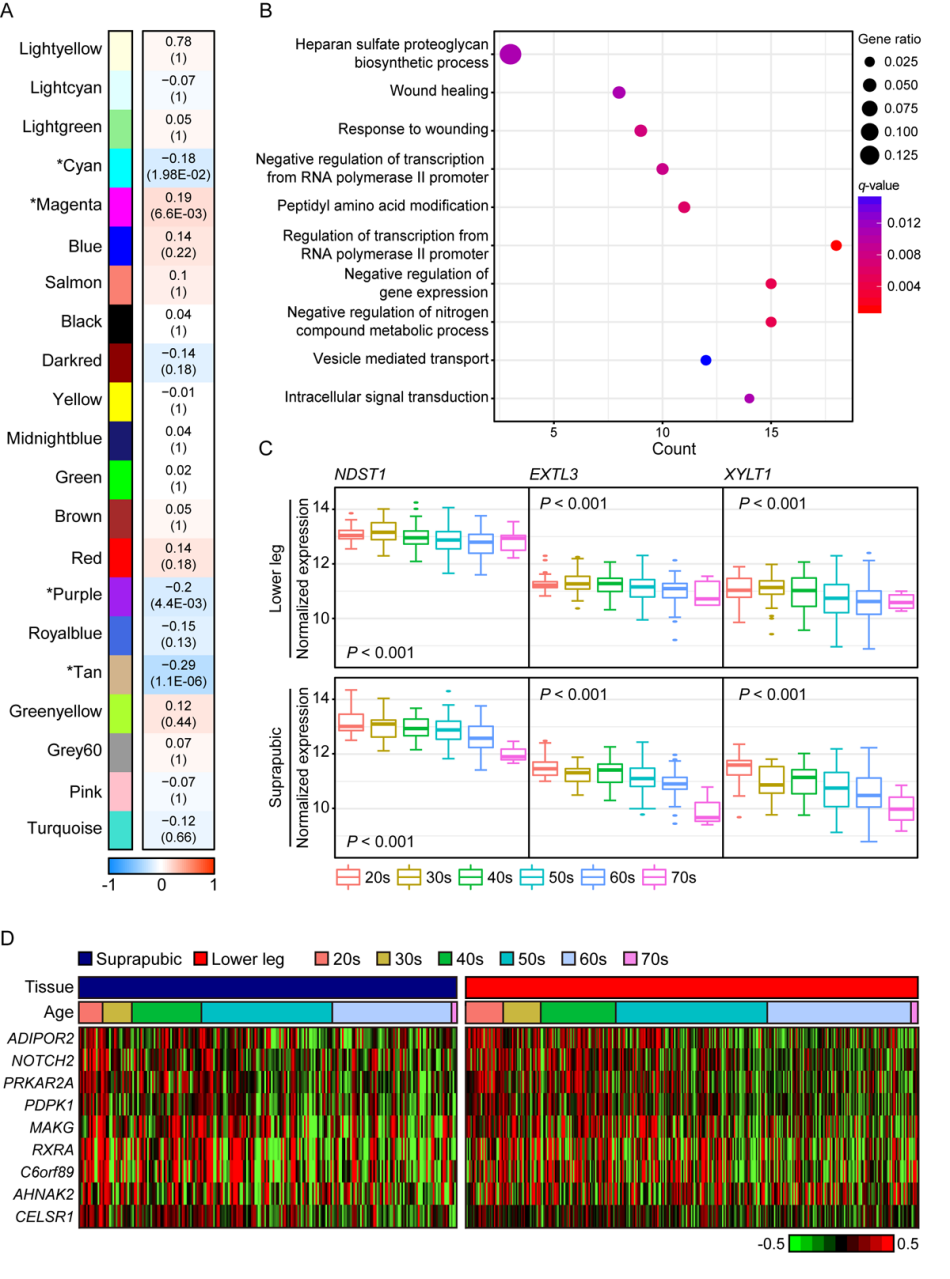
**The common gene expression changes with aging in suprapubic and lower leg tissues.** (**A**) Module trait relationship of constructed modules from lower leg. The statistically significant modules were marked with asterisk. The correlation values and Bonferroni-corrected *P*-values (in the bracket) were marked. (**B**) Pathway enrichment analysis of tan module. Top 10 the most enriched pathways were used. (**C**) The changes in gene expression level of three genes that play a role in heparan sulfate proteoglycan biosynthetic process in both tissues were shown. (**D**) Heatmap displays the expression level of nine genes that play a role in wound healing process in both tissues.

We also presented photo-aging signatures by performing WGCNA using only lower leg skin samples and 21 aging-related modules were constructed and four of them were statistically significant (Figure 3A). In addition, the top 10 genes with the highest connectivity from four significant modules are listed on Supplemental Table 2. The most correlated module was tan (R = -0.29, Bonferroni-corrected *P* = 1.1E-06) and it was mainly enriched with wound healing related pathways including ‘heparan sulfate proteoglycan biosynthetic process’, ‘wound healing’, and ‘response to wounding’ (Figure 3B). Heparan sulfate is a type of glycosaminoglycans, a group of long-chain carbohydrates with high molecular weight, that are sulfated [33] and proteoglycans play crucial roles in regulating several physiological processes including wound healing [34]. We found three genes, *EXTL3*, *NDST1*, and *XYLT1*, that were involved in ‘heparan sulfate proteoglycan biosynthetic process’ displayed decrement with aging in both tissues (Figure 3C). In particular, *NDTS1* was one of top 10 most correlated genes found in this module (Supplemental Table 2). Also, there were nine genes discovered that were enriched in ‘wound healing’ and ‘response to wounding’ (Figure 3D). Those genes were also found in modules which were associated with aging in suprapubic (red and yellow modules; Supplemental Figure 2).

**Figure 4:**
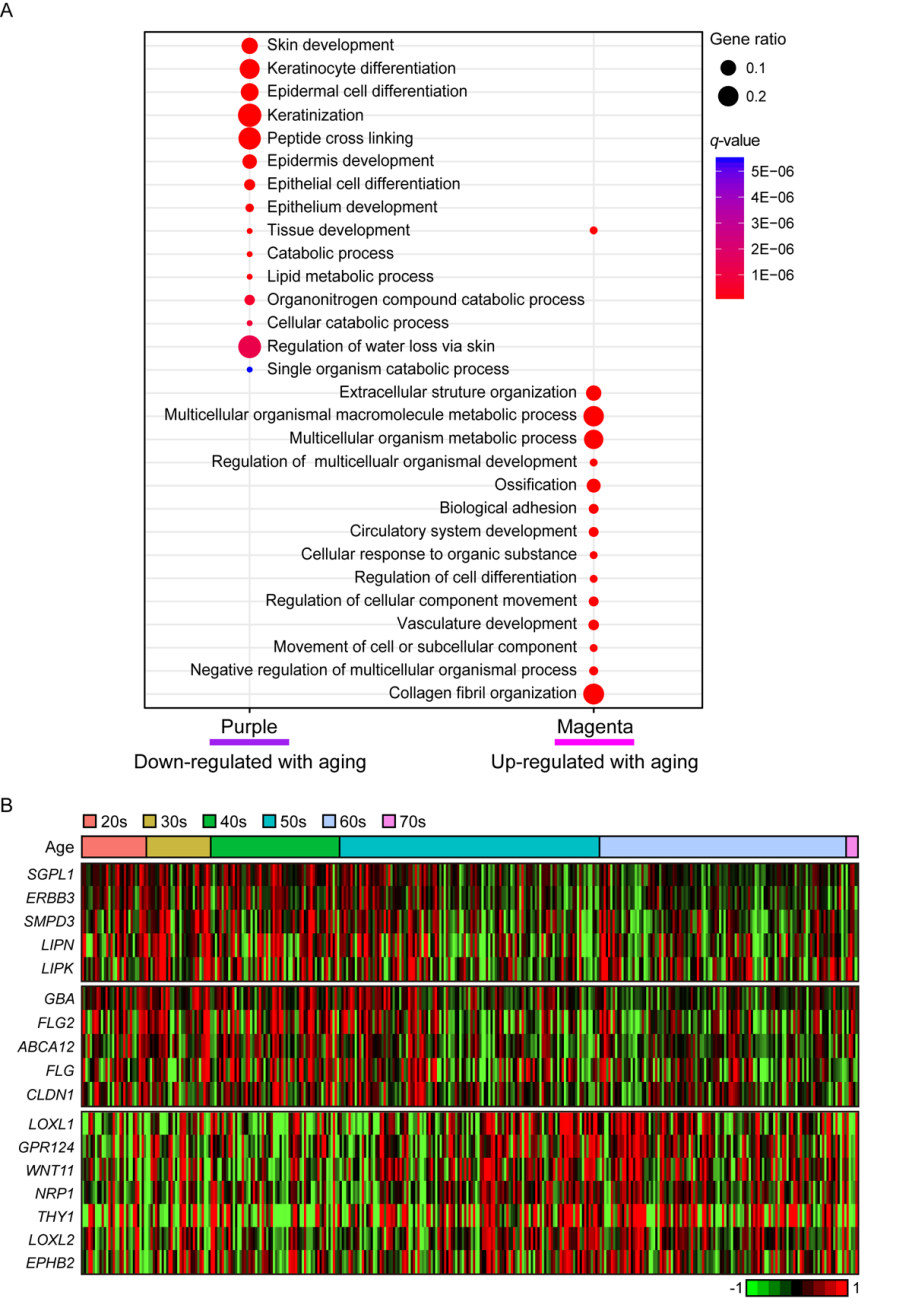
**The gene expression alterations that are specific to lower leg.** (**A**) Pathway enrichment analysis of purple and magenta modules. Top 15 the most enriched pathways were used. (**B**) Heatmap displays the gene expression changes with aging involved in lipid metabolism (top), water loss regulation via skin (middle), and vasculature development (bottom) in lower leg.

In purple module (R = -0.2 and Bonferroni-corrected *P* = 4.4E-03), ‘skin/tissue development’, ‘lipid metabolic process’, and ‘regulation of water loss via skin’ were found (Figure 4A). Lipid metabolism and prevention of water loss are known to have roles in skin barrier function and often influenced by aging [35,36]. Genes involved in lipid metabolism such as *ERBB3*, *LIPK*, *LIPN*, *SGPL1*, and *SMPD3* were decreased with aging as well as several water loss regulation genes including *ABCA12*, *CLDN1*, *FLG*, *FLG2*, and *GBA* (Figure 4B). Aforementioned genes were not found in statistically significant aging-related module in suprapubic (yellow, salmon, and pink modules; Supplemental Figure 2). In addition, 12 LCE genes were detected as down-regulated with aging (Supplemental Figure 3). However, from suprapubic, those genes were enriched in salmon module which did not change statistically significant with aging (Supplemental Figure 2).

Moreover, magenta module (R = 0.19 and Bonferroni-corrected *P* = 6.6E-03) was up-regulated with aging (Figure 4A). Intriguingly, ‘vasculature development’, which also showed increment with UV exposure, may be related to wrinkle generation, displayed aging-related up-regulation in lower leg. Well-known angiogenesis genes including *NRP1, LOXL1* and *THY1* were enriched in this pathway (Figure 4B) [37–41]. Especially, *THY1* and *LOXL1* were found as top 10 most correlated genes in magenta module (Supplemental Table 2). However, the same pathway did not show clear down-regulation in suprapubic (darkred, turquoise, and tan; Supplemental Figure 2). Also, in magenta, collagen related pathways were found, for instance ‘extracellular structure organization’ [42] (Figure 4A). Here, a number of collagen genes including *COL5A1*, *COL6A1*, and *COL13A1* were elevated which was only observed in lower leg, not in suprapubic (Supplemental Table 4).

Lastly, genes from cyan module (R = -0.18 and Bonferroni-corrected *P* = 1.98E-02) were decreased with aging in lower leg. ‘Molting cycle’, ‘skin/epidermis development’, and ‘aging’ were enriched and 69% of total analyzed keratin genes were discovered in this module (Supplemental Figure 4 and Supplemental Table 5). Those keratin genes were also found in tan module (R = -0.09, Bonferroni-corrected *P* = 1), constructed from suprapubic, however they did not show significant elevation with aging.

## DISCUSSION

Every organ experiences aging in various ways. Skin aging can be categorized into three groups, intrinsic, photo, and hormonal aging [43–45]. In recent years, the interests in revealing the cause of aging in transcriptome level and how to prevent aging are increasing [3–6,46–48]. In the present study, we primarily focused on finding the consequences of intrinsic- and photo-aging of skin. Currently, there are several studies defined the consequence of acute UV exposure using *in vivo* or *in vitro* system whereas there are not many studies on finding the effect of chronic UV exposure, especially by using human tissue samples [27,49–51]. Thus, performing in-depth analysis using human skin samples enables us to describe actual biological changes by UV exposure, hence may help to utilize the findings in our day-to-day life.

In our analyses, down-regulated lipid metabolism and up-regulated vasculature development were highlighted as the characteristics of photo-aged skin. Moreover, decreased mitochondrial biogenesis and wound healing process were found as features of aged skin. In addition to aging, we elucidated the traits of sun exposed skin and discovered increased expression of EDC genes and *MMP* genes upon UV exposure.

Aging induces the decline of various body functions and lipid metabolism is one of them [52]. There are a few reported studies describing the correlation between decreased lipid metabolism with skin aging [22,23]. Lipid metabolism contributes to the protective function of skin and impaired lipid metabolism compromises the barrier function of the skin [35,53]. In our result, ‘lipid metabolic process’ was remarkably decreased with photo-aging. Especially, genes that have role in epidermal barrier function including *LIPN*, *LIPK*, and *SMPD3* showed dramatic decreased with photo-aging [24].

The consequences of photo-aging are usually characterized by morphological changes including wrinkle formation or by histological changes in connective tissues [29,30,54,55]. Increased wrinkle formation is the most common phenomenon of aging [56]. In order to generate more wrinkles, vasculature structures are needed to develop. Numerous reports suggested that angiogenesis plays a significant role in inducing wrinkle formation in photo-damaged skin [28,49,54,57,58]. In our result, we discovered that ‘vasculature development’ was increased with photo-aging. Genes that functions critically in this process including *TGFBR1*, *TGFBR2*, *TGFBR3*, *KDR*, *FGF2*, and *VEGFC* showed a trend of increment with photo-aging.

The trait of aged tissue can be described by showing declined mitochondrial biogenesis [59]. For several decades, many scientists suggested the involvement of mitochondria in aging process by showing mitochondrial dysfunction or mtDNA damage with aging [60–62]. In this study, we also demonstrated the correlative data displaying decreased mtDNA copy number with aging in UV-protected and -exposed skin. A clear trend of decrement was exhibited with aging which supported previous studies.

Another decreased biological process due to aging in skin was ‘wound healing’. The correlation between weakened wound healing process and aging was previously reported [33,63–65]. In particular, an *in vivo* study demonstrated that the rate of wound healing was delayed 20% to 60% with aging [63]. Here, we illustrated the decrement of wound healing related genes including *ADIPOR2*, *NOTCH2*, *PRKAR2A*, *PDPK1*, *MAKG*, *RXRA*, *C6orf89*, *AHNAK2*, and *CELSR1* with photo-aging together with intrinsic-aging. Moreover, three key genes, *NDST1*, *EXTL3*, and *XYLT1*, that play roles in ‘heparan sulfate proteoglycan biosynthetic process’ which is involved in wound healing also exhibited decrease with aging in UV-protected and -exposed skin which strengthened our result.

In addition, we identified the increased expression level of EDC genes in photo-damaged skin which play an important role in epidermis maturation [16,66]. Jackson *et al*. described that group 3 *LCE* genes showed magnificent elevation upon acute UV exposure, especially *LCE3C* which also displayed similar trend in our analysis. We have also presented that increased levels of many EDC genes including *LCE*s, *SPRR*s, and *FLG* with UV exposure. Additionally, it is well-known that several *MMP* genes are up-regulated in photo-damaged skin [14,67]. Quan *et al*. and Waldera *et al*. showed increment of *MMP* genes with UV exposure such as *MMP2*/*3*/*9*/*11* and *MMP1*/*3*/*10*/*14*, respectively. These findings corresponded to our result as *MMP2*, *MMP3*, *MMP8*, *MMP10*, and *MMP13* also displayed increased expression level in photo-damaged skin. Increased expression of *MMP* genes disrupt collagen structure in skin which then leads to impaired skin condition [68]. Interestingly, in our result, a few collagen genes showed up-regulation which suggests that there are a lot more to explore in regards to collagen synthesis and aging.

The era of big data has opened its chapter and biological research using big data can strengthen the outcome in various ways. Sometimes, in experimental research, an experiment is usually performed targeting single or a small number of genes only. Assigning a small number of genes to in-depth study may miss the fact that the functional result of assigned genes might be involved in more than one pathway. As much as the validation of a gene’s biological function is important, transcriptome research, especially with co-expression network analysis, allows us to investigate a group of genes with similar function that regulate a biological pathway in question. Therefore, with aid of experimental data, we believe that our study may further expand the understating of human aging process and facilitate the development of therapeutic targets for skin rejuvenation.

## MATERIALS AND METHODS

### Study subjects

GTEx database (V6 release) provided 606 skin samples from suprapubic (n = 250) and lower leg (n = 356) [7,8]. The age groups are from 20s to 70s and they are from both genders and all skin tissue samples were obtained by rapid autopsy from donors. Among them, 22 samples with hair or hair follicles were disregarded to the analysis. Also, seven samples with solar elastosis were eliminated to the analysis to focus on the gene expression changes of normal skin. Therefore, 228 suprapubic and 349 lower leg samples were used (Table 1).

**Table 1:** Sample information.

**Tissue type**	**20s**	**30s**	**40s**	**50s**	**60s**	**70s**	**Total**
Suprapubic (UV protected)	14 (6.14%)	18 (7.89%)	42 (18.42%)	79 (34.65%)	72 (31.58%)	3 (1.32%)	228
Lower leg (UV exposed)	29 (8.31%)	29 (8.31%)	58 (16.62%)	117 (33.52%)	111 (31.81%)	5 (1.43%)	349

### Differentially expressed gene analysis

We discovered differentially expressed genes (DEGs) using DESeq2 [69]. Here, genes with *q*-value < 0.05, |Log_2_(fold-change)| ≥ 0.6, and baseMean ≥ 100 were determined as DEGs. Multiple testing was performed by Benjamini-Hochberg method [70].

### Weighted gene co-expression network analysis

Weighted gene co-expression network analysis (WGCNA) identified the associations of the gene expression changes in skin with UV exposure status or aging [71]. The variance stabilizing transformed expression values from DESeq2 were used for the analysis [69,72]. Briefly, pairwise correlations between the expression of each gene were used to construct modules which denote the co-expression network. The module eigengene is characterized as the first principal component of each module. To identify the relationship between the modules and the clinical characteristics, the correlations between module eigengenes and clinical traits were determined using the Pearson correlation coefficient. For determining the significance of modules generated from WGCNA, Bonferroni-corrected *P*-values were used [73]. Using module membership (MM) values, which indicate the high intra-module connectivity within each module, top 10 most correlated genes from each module were listed on Supplemental Tables.

### Pathway enrichment analysis

The pathway enrichment analysis computed biologically meaningful pathways using the Molecular Signatures Database (MSigDB) version 6.0 [74]. The gene ontology (GO) biological process database from MSigDB was used in this study [74]. To illustrate the pathway enrichment result, clusterProfiler was used [75]. The significance was determined by the color using *q*-value and the gene ratio stands for the ratio of enriched genes in each gene set.

### Mitochondrial DNA copy number estimation

To estimate mitochondrial copy number, 16 mitochondrial genes (*MT-RNR1*, *MT-RNR2*, *MT-ND1*, *MT-ND2*, *MT-CO1*, *MT-TS1*, *MT-CO2*, *MT-ATP8*, *MT-ATP6*, *MT-CO3*, *MT-ND3*, *MT-ND4*, *MT-ND4L*, *MT-ND5*, *MT-ND6*, *MT-CYB*) with the average mapped reads higher than 100 were subjected to the analysis. We calculated mitochondrial copy number by averaging 16 mitochondrial RNA (mtRNA) expression values. The association between aging and mtRNA expression were measured by one-way ANOVA and test for Linearity using IBM SPSS Statistics Version 23.0.

## SUPPLEMENTARY MATERIAL

Supplemental Figure 1

Supplemental Figure 2

Supplemental Figure 3

Supplemental Figure 4

Supplemental Table 1

Supplemental Table 2

Supplemental Table 3

Supplemental Table 4

Supplemental Table 5

Supplemental Table 6
