# Supplementary figures and images for: Signatures of photo-aging and intrinsic aging in skin were revealed by transcriptome network analysis
Source: Aging (Albany NY). 2018 Jul 18;10(7):1609–26. doi: 10.18632/aging.101496 (PMC6075446; doi:10.18632/aging.101496)

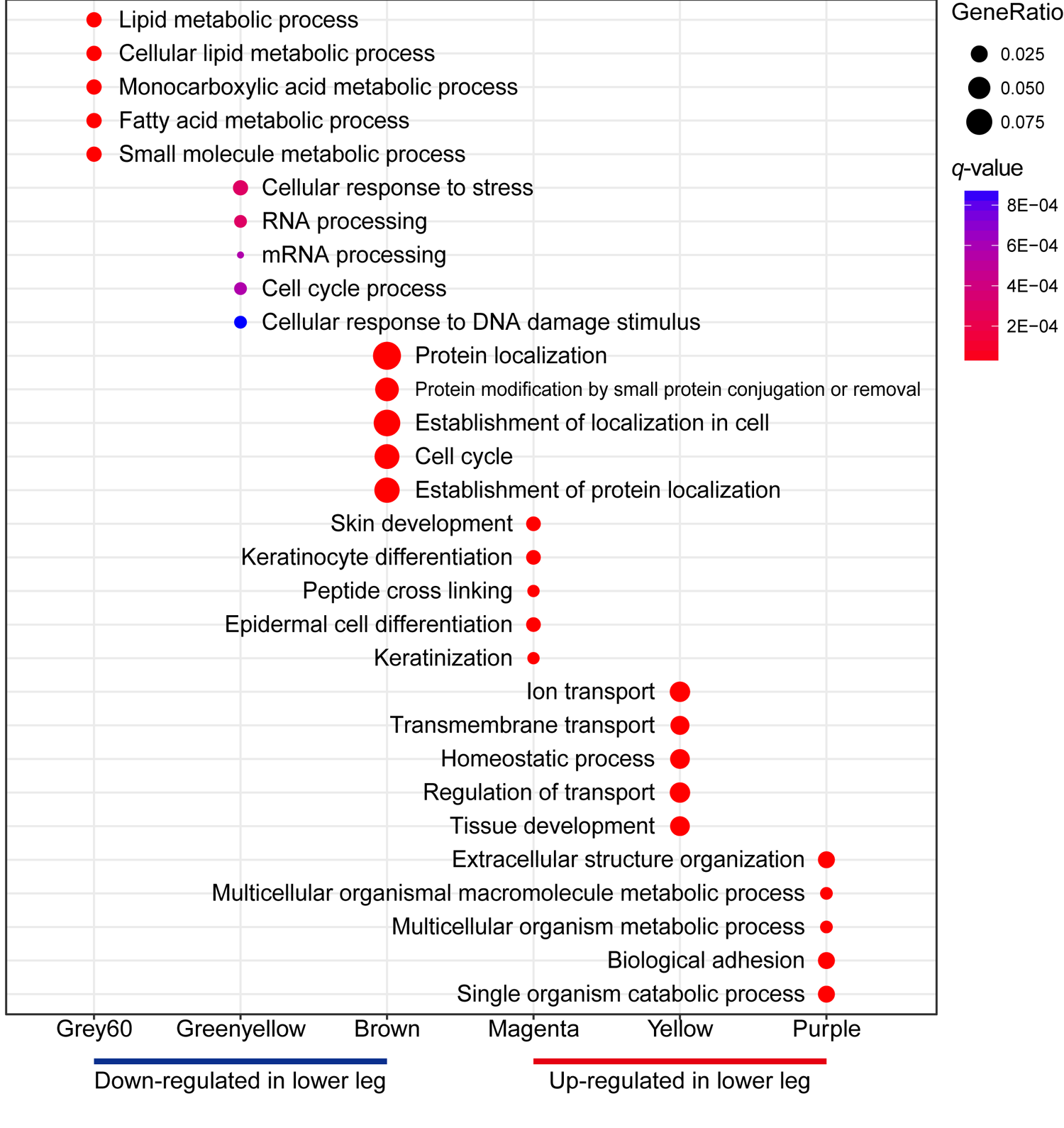

Supplement: Supplemental Figure 1 [file aging-10-101496-s001.tif]

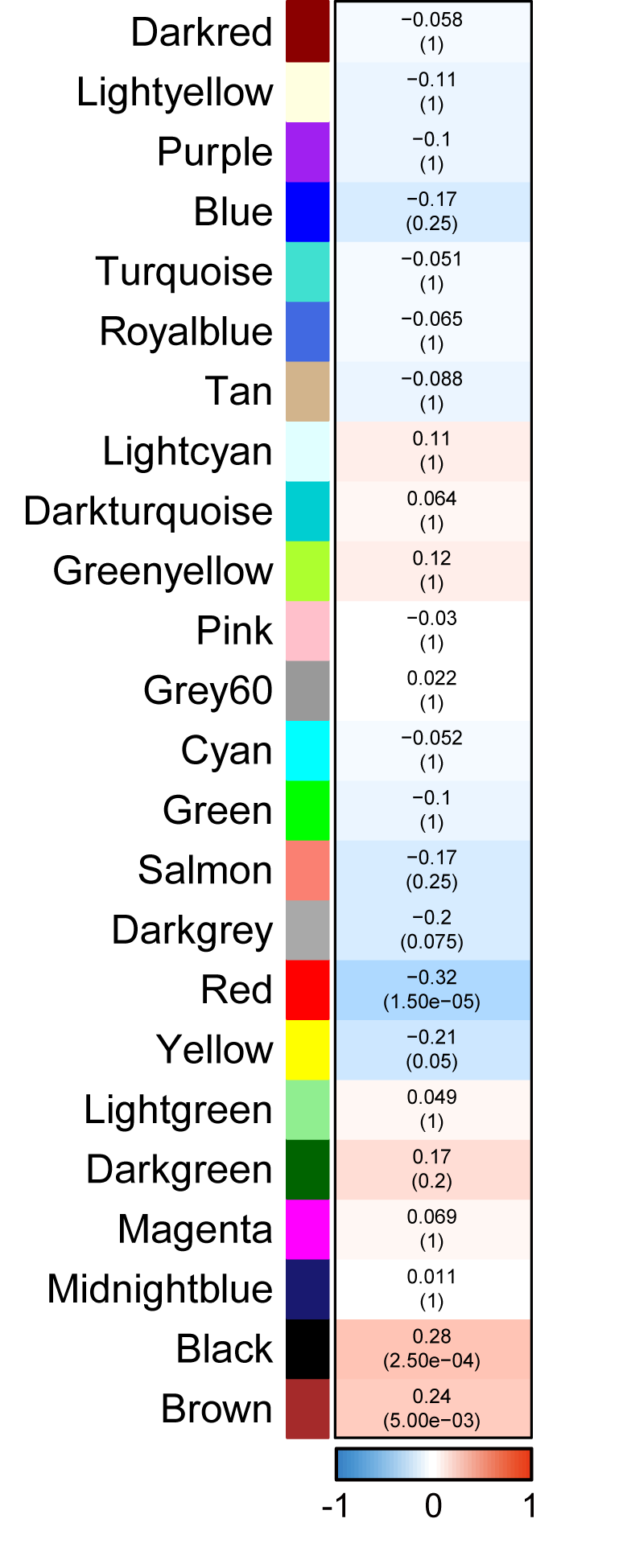

Supplement: Supplemental Figure 2 [file aging-10-101496-s002.tif]

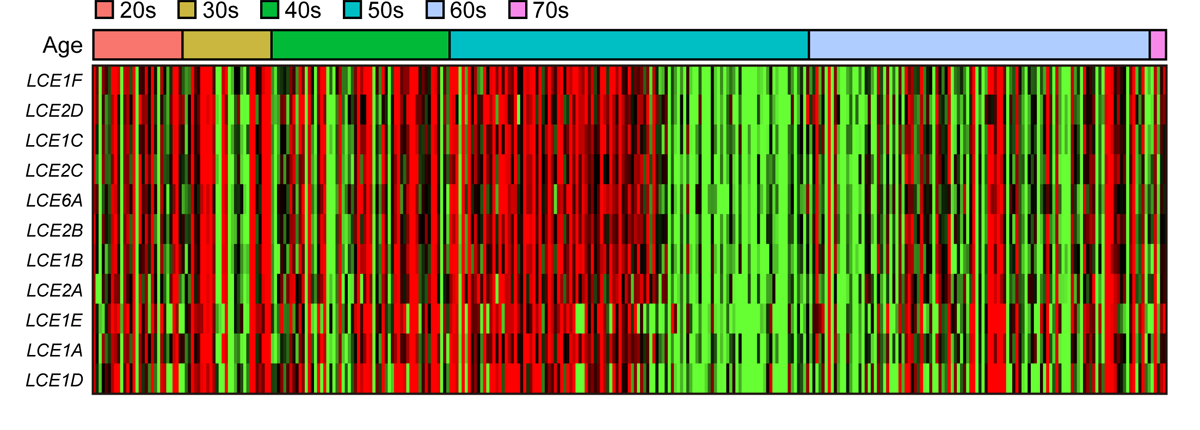

Supplement: Supplemental Figure 3 [file aging-10-101496-s003.tif]

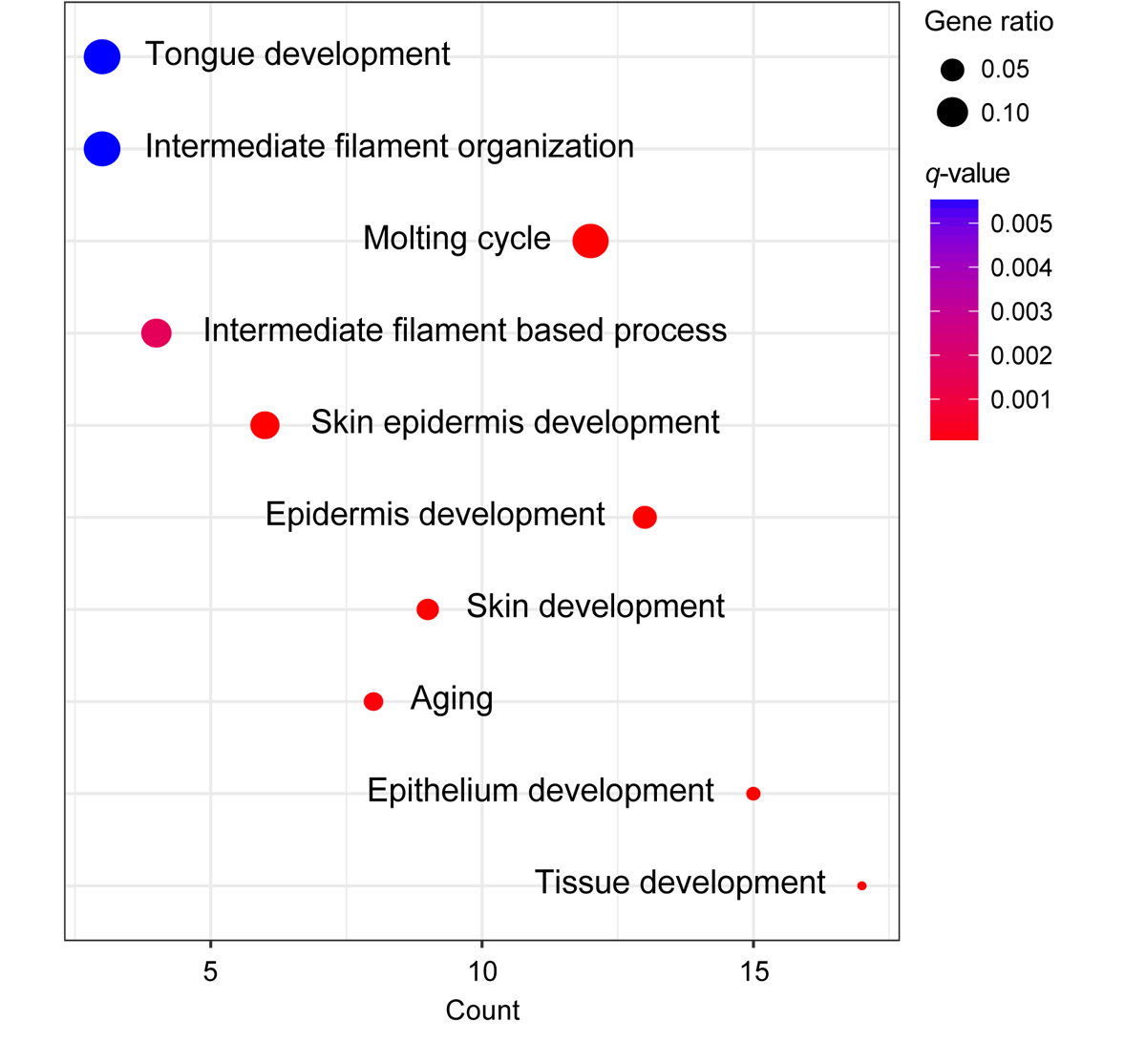

Supplement: Supplemental Figure 4 [file aging-10-101496-s004.tif]
